# Preference for and Discrimination of Paintings by Mice

**DOI:** 10.1371/journal.pone.0065335

**Published:** 2013-06-06

**Authors:** Shigeru Watanabe

**Affiliations:** Department of Psychology, Keio University, Mita 2-15-45, Minato-Ku, Tokyo, Japan; Duke University, United States of America

## Abstract

I measured preference for paintings (Renoir vs. Picasso or Kandinsky vs. Mondrian) in mice. In general mice did not display a painting preference except for two mice: one preferred Renoir to Picasso, and the other preferred Kandinsky to Mondrian. Thereafter, I examined discrimination of paintings with new mice. When exposure to paintings of one artist was associated with an injection of morphine (3.0 mg/kg), mice displayed conditioned preference for those paintings, showing discrimination of paintings by Renoir from those by Picasso, and paintings by Kandinsky from those by Mondrian after the conditioning. They also exhibited generalization of the preference to novel paintings of the artists. After conditioning with morphine for a set of paintings consisting of two artists, mice showed discrimination between two sets of paintings also from the two artists but not in association with morphine. These results suggest that mice can discriminate not only between an artist’s style but also among paintings of the same artist. When mice were trained to discriminate a pair of paintings by Kandinsky and Renoir in an operant chamber equipped with a touch screen, they showed transfer of the discrimination to new pairs of the artists, but did not show transfer of discrimination of paintings by other artists, suggesting generalization.

## Introduction

A recent study of ancient cave paintings demonstrated that humans have been painting for more than 40,000 years [1]. Art and aesthetics seem to be unique human abilities. Aesthetics has two aspects. One is the cognitive aspect. People can discriminate painting style and also discriminate “good” or “beautiful” paintings from “bad” or “ugly” paintings; they have a sensory concept or category of “good” painting. The second aspect is the hedonic or pleasure aspect. Looking at good art brings pleasure to humans; in other words, art has reinforcing properties in humans.

Rensch [2] [3] examined preferences for visual patterns in several species. Using capuchin monkeys, he described the primates’ preference for regular, symmetrical patterns. Crows and meerkats also show a preference for regular patterns, but fish do not. Several experimental studies have demonstrated the reinforcing effect of complex visual stimuli in non-human animals [4] [5], but such a reinforcing effect of paintings by professional artists has rarely been examined. Ikkatai and Watanabe [6] examined the preference for paintings (cubist, impressionist, and Japanese) by Java sparrows and found that six of seven birds preferred cubist paintings to impressionist paintings. The birds could also discriminate these paintings in conventional operant discrimination training with a food reward. Pigeons can discriminate paintings by Monet from those by Picasso [7], paintings by Chagall from those by Van Gogh [8], and Japanese paintings from impressionist paintings [9]. I have also shown discrimination of “good” and “bad” children’s paintings by pigeons [9] [10]. These studies demonstrate that human visual art has discriminative and reinforcing properties in some birds. One similarity between humans and birds is their highly developed visual system, which may be the basis of such behavior.

Contrary to birds, rodents are generally considered non-visual animals, but mice use visual cues for social cognition [11]. Mice may use fine visual cognition to discriminate complex non-social visual stimuli. Here, I examined painting preference and painting discrimination in mice using association with a reinforcing drug. I used paintings by Kandinsky and Mondrian (both are abstract painters) in the first series of experiments, and then paintings by Renoir (impressionist) and Picasso (cubist) in the second series of experiments. For experiment 1, I tested preference between the paintings by two artists and then examined conditioned painting preference in which paintings of one of the two artists were associated with morphine injection. I also tested generalization of the conditioned preference to novel paintings by the same artist. I hypothesized that all paintings by one artist, for example Picasso, might look alike to mice. To examine this possibility, I randomly classified paintings by two artists into two sets, with each set containing paintings from both artists, then I conditioned mice to associate one set of paintings with the drug, but not the other set. Finally, I trained mice using operant discrimination of paintings with a food reward in experiment 2.

## Results

### Experiment 1: Spontaneous Preference and Conditioned Preference

**Figure 1:**
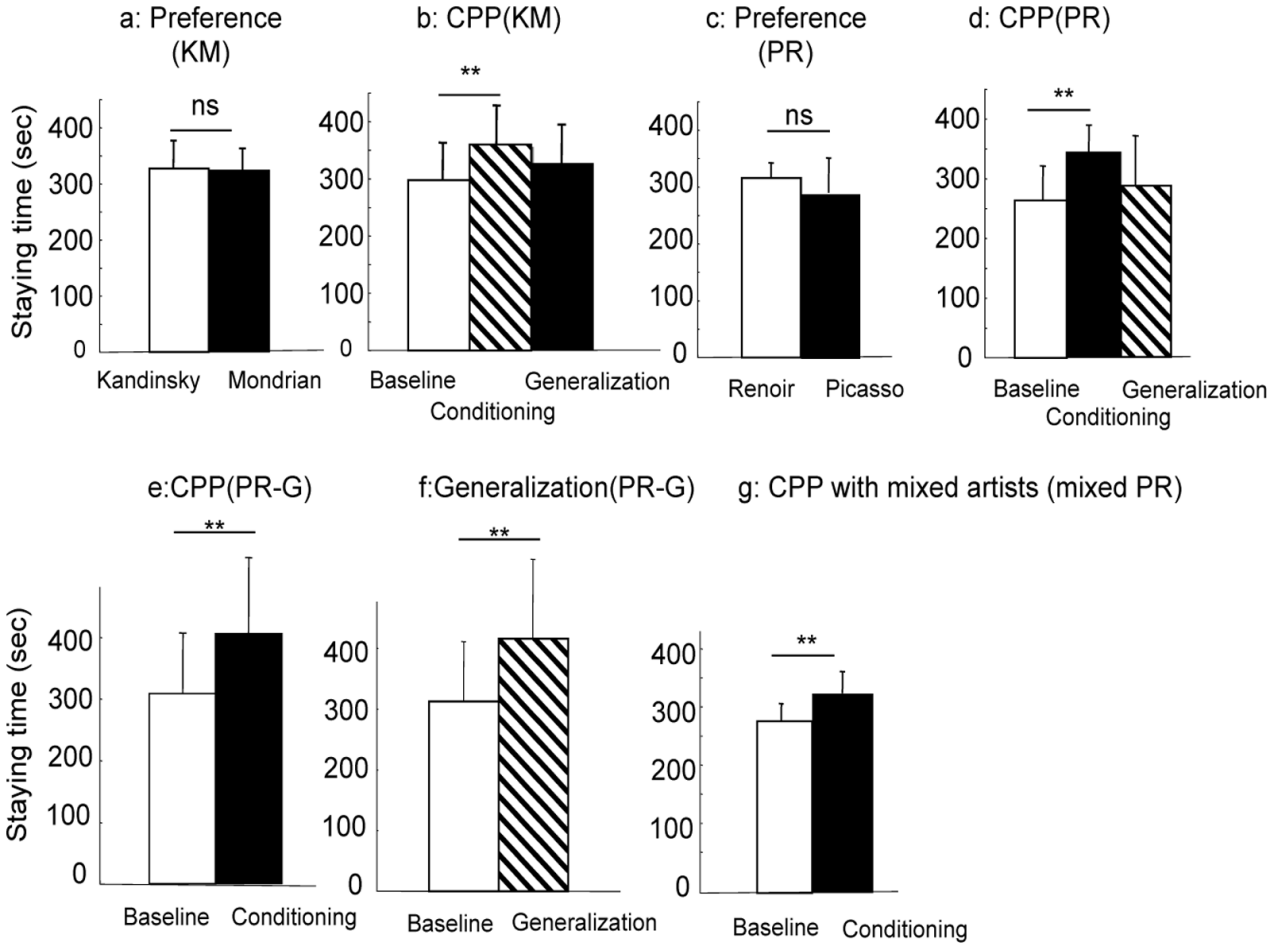
Results of experiment 1. a: Preference test with paintings by Kandinsky and Mondrian, b: Conditioned painting preference with Kandinsky and Mondrian, c: Preference test with paintings by Picasso and Renoir, d: Conditioned painting preference with Picasso and Renoir, e: Conditioned painting preference with 5 paintings by Picasso and Renoir, f: Generalization test with another 5 paintings by Picasso and Renoir, g: Conditioned painting preference with mixed artists. ns = not significant, ** *P*<0.05. KM, PR,PR-G and mixed PR indicate Kandinsky-Mondrian group, Picasso-Renoir group, Picasso-Renoir generalization group and mixed Picasso-Renoir group respectively.

Figure 1a shows the staying time in the compartment with paintings by Kandinsky and Mondrian. In the preference test, the mice (N = 20) stayed in the two compartments for approximately equal amounts of time. The paired *t*-test revealed no significant difference in staying time between the two sides (*t*(19) = 0.06, *P* = 0.95). Thus, the mice did not show a preference between Kandinsky and Mondrian. Analysis of individual mice revealed only one mouse out of 20 mice displayed some preference for Kandinsky during 6 days of the test (*t*(5) = 2.53, *P* = 0.053), suggesting the rare possibility of picture preference in mice.

Figure 1b shows the mean staying time in the compartment with paintings associated with morphine injection before and after conditioning (N = 20). The two-factor ANOVA (artists × conditioning) showed a significant effect of conditioning (*F*(1/39) = 5.73, *P* = 0.02) but no significant effect of artists (*F*(1/39) = 0.68, *P* = 0.41) and no interaction (*F*(1/39) = 0.15, *P* = 0.70). There was a significant difference in staying time between the baseline and the test (paired *t*-test, *t*(19) = 3.20, *P* = 0.005). Thus, the mice preferred the paintings associated with morphine injection to the other paintings. The mice stayed longer in the compartment with the novel paintings of the artist associated with morphine injection in the generalization test in comparison to the baseline but less than at the conditioning test. Statistical analysis showed a tendency toward a difference between the baseline and the generalization test (*t*(19) = 1.73, *P* = 0.09) but no significant difference between the post-conditioning test and the generalization test (*t*(19) = 1.72, *P* = 0.10). These analyses suggest there was generalization to some degree, but the phenomenon could not be statistically validated.

Figure 1c shows the mean staying time at paintings by Renoir and Picasso in the preference test (N = 12). The mice stayed slightly longer in the compartment with Renoir paintings, but the difference was not statistically significant (paired *t*-test, *t*(11) = 1.80, *P* = 0.09). Individual analysis showed that one mouse significantly preferred paintings by Renoir during 6 days of the preference test (*t*(5) = 10.65, *P* = 0.0001).

Figure 1d shows the staying time in the compartment associated with morphine injection before and after conditioning using paintings by Picasso and Renoir. The mice clearly stayed longer after the conditioning (N = 12). Two-factor ANOVA (artists × conditioning) revealed a significant effect of the conditioning (*F*(1/23) = 14.36, *P* = 0.001), but no significant effect of the artists (*F*(1/23) = 0.66, *P* = 0.42) or interaction (*F*(1/23) = 1.50, *P* = 0.24). The paired *t*-test showed a significant difference in staying time before and after the conditioning (*t*(11) = 4.34, *P* = 0.001). The mice stayed longer in the compartment with paintings of conditioned artists in the generalization test, but there was no significant difference between baseline and generalization (*t*(11) = 1.05, *P* = 0.32), whereas there was a significant difference between the post-conditioning test and the generalization test (*t*(11) = 2.92, *P* = 0.014). Thus, the mice did not show clear generalization to the novel paintings.

There was no baseline preference data for the paintings used for the generalization test in these experiments. In the next experiment (N = 14), I divided 10 paintings by Picasso and Renoir each into two groups of 5 and measured baseline preference for each group and then presented 5 paintings of Picasso and Renoir during the conditioning. In generalization test after the conditioning, I presented the paintings used for conditioning to examine the effect of the conditioning and also another 5 paintings never shown during the conditioning. Figure 1e shows the staying time compartment associated with morphine injection before and after conditioning. Two-factor ANOVA (artists × conditioning) revealed a significant effect of the conditioning (*F*(1/27) = 6.90, *P* = 0.01) but no significant effect of the artists (*F*(1/27) = 0.0002, *P* = 0.99) or interaction (*F*(1/27) = 0.13, *P* = 0.72). There was a significant difference between the baseline and test (*t*(13) = 2.83, *P* = 0.01) demonstrating conditioned preference for the paintings associated with morphine injection. Figure 1f shows the staying time in the compartment with the paintings used for the generalization test before and after conditioning. The mice exhibited an increment in the staying time after the conditioning. Two-factor ANOVA (artists × conditioning) revealed a significant effect of the conditioning (*F*(1/27) = 5.24, *P* = 0.03), but no significant effect of the artists (*F*(1/27) = 0.17, *P* = 0.69) or interaction (*F*(1/27) = 1.73, *P* = 0.20). There was a significant difference between the baseline and test (*t*(13) = 2.16, *P* = 0.05). Thus, the mice displayed a generalization effect of conditioning on the stimuli which were never used for the conditioning.

As noted above, there is a possibility that all paintings by one artist look the same for mice. To examine this possibility, I randomly classified paintings by Picasso and Renoir into two sets, with each containing both artists, and I conditioned mice to associate one set with the drug, but not the other set. Figure 1g shows the staying time in the compartment with paintings associated with morphine injection before and after conditioning. The mice (N = 12) stayed longer in the drug-associated compartment after the conditioning. There was a significant difference between the baseline and test (*t*(11) = 3.27, *P* = 0.007). These results clearly showed discrimination among Renoir’s paintings and among Picasso’s paintings.

### Experiment 2: Operant Discrimination

**Figure 2:**
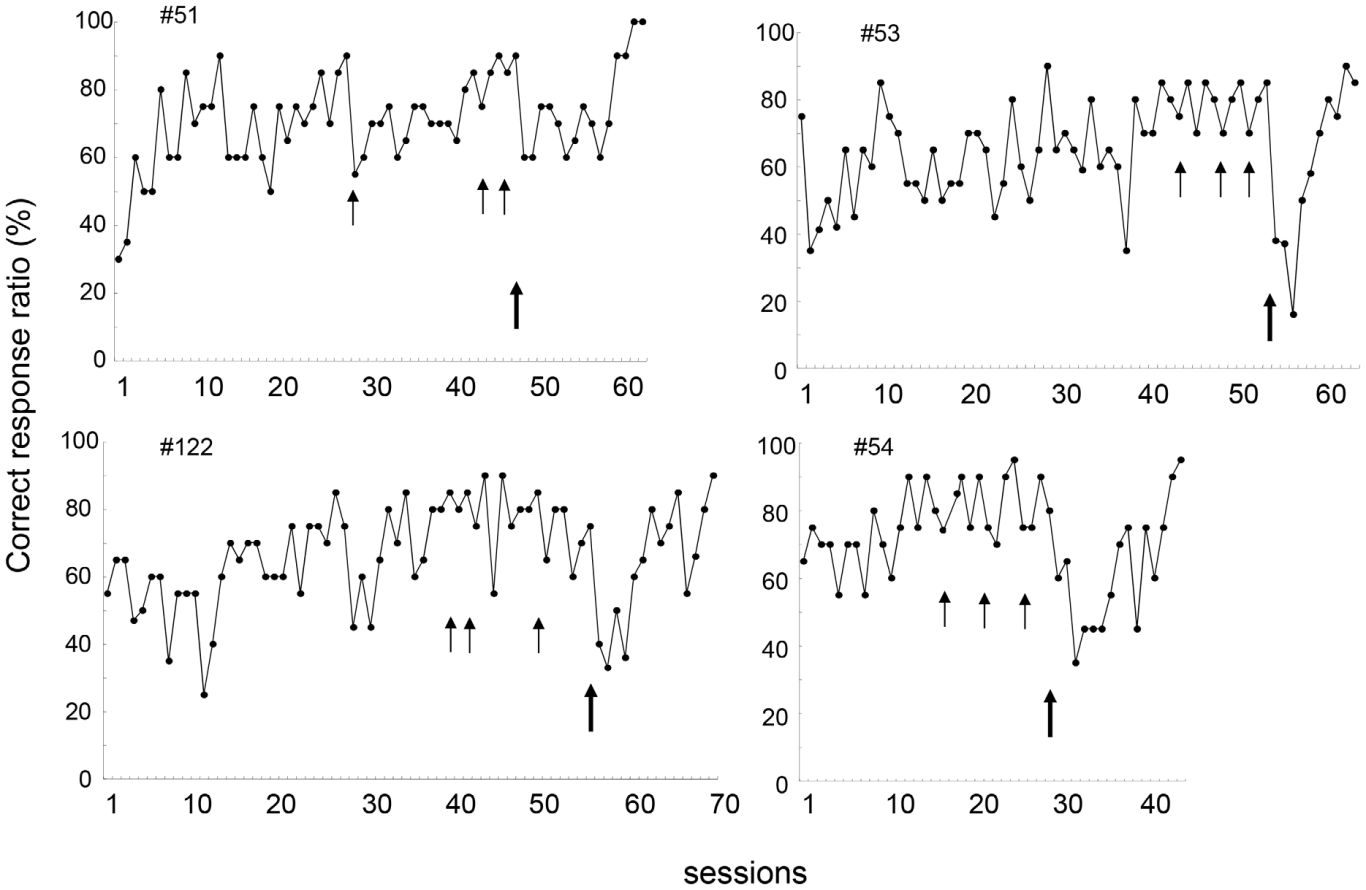
Operant discrimination of paintings by Kandinsky and Mondrian by four mice. The correct response ratio was obtained by dividing the number of response to S+ by the total number of trials. Arrows indicate the start of training with a new pair of paintings. The last bold arrows indicate a pair of Picasso and Renoir.

Figure 2 shows the results of operant discrimination between paintings by Kandinsky and those by Mondrian. Small arrows indicate start of a discrimination task with new pair of paintings by Kandinsky and Mondrian. The fastest mouse required 16 sessions and the slowest 43 sessions (average 31.5 sessions) to learn the first task. To learn the fourth task, the fastest mouse needed 2 sessions and the slowest 4 sessions. The correct response ratio in the first session of the second task was 0.55 to 0.85 (average 0.74) and that in the fourth task was 0.70 to 0.85 (average 0.79). Thus, the mice were able to discriminate paintings and transfer the discrimination to novel stimuli. When they saw a pair of Picasso and Renoir (indicated by bold arrows), the mice did not show transfer of discrimination. The correct response ratio in the first session was 0.50 to 0.60 average 0.57). Paired *t*-test revealed a significant difference in correct discrimination ratio in the first session between the last Kandinsky-Mondrian discrimination task and the Picasso-Renoir discrimination task (*t*(3) = 8.88,*P*<0.005). They were able to learn the new task but required 10 to 15 sessions (average 13.5 sessions). There was a significant difference in number of sessions to reach the criterion between the last Kandinsky-Mondrian discrimination task and the Picasso-Renoir discrimination task (*t*(3) = 8.20,*P*<0.005). These results demonstrate within artist transfer and no cross artist transfer suggesting generalization within the artist.

## Discussion

The present results demonstrate that painting preference is quite rare, if it exists at all, in mice, but that they can discriminate the paintings. To my knowledge, this is the first report of painting discrimination in rodents, suggesting that mice possess acute visual cognition than previously believed. Previously, I used Conditioned Place Preference (CPP) paradigm for individual discrimination in mice and quails in which presentation of one particular stimulus animal was associated with injection of morphine into the subject animal [12] [13]. The present results also extended the application of CPP paradigm in investigations of complex visual discrimination. Because CPP requires short training sessions compared to operant conditioning, it is an efficient technique for analyzing discriminative behavior in rodents.

Mice also showed generalization to novel paintings after the conditioning, and results of the operant discrimination supported the possibility of generalization. Because they successfully learned to discriminate two groups of mixed Renoir/Picasso paintings, they also likely discriminated each painting, implying that category or concept discrimination of paintings may exist in mice. Animals can discriminate any stimuli if the stimuli are sufficiently psychophysically different. Renoir and Picasso, or Mondrian and Kandinsky, may pose as distinct stimuli for mice. The discrimination displayed here would seem to represent discrimination of meaningless visual patterns in mice. Although we found representation of objects in paintings by Renoir, these cues were mostly difficult to find in paintings by Picasso and impossible to detect in paintings by Mondrian or Kandinsky. Accordingly, the precise crucial cues for discrimination are not known in the present experiment and warrant further investigations.

Painting preference was not observed in 32 mice and was only seen in two mice. The reinforcing property of music is also quite difficult to observe in non-human animals. Currently, only humans and songbirds are known to display the reinforcing property of music [14]. Monkeys [15], rats [16], pigeons [17], and goldfish [18] do not show music preferences, but these species may be able to discriminate music. Both humans and songbirds can participate in complex auditory communication, and reinforcing properties of auditory stimuli may contribute to this phenomenon. Comparative data of reinforcing properties of paintings are too limited to draw any conclusions, but fine visual cognition is a prerequisite for the reinforcing property of visual art. As I pointed out in Introduction, mice use visual cues for social cognition [11] and they may apply this visual ability to discriminate paintings. In comparison to similarity between bird song and human music, similarity of visual stimuli for social communication in mice and paintings in humans is quite low. This could be one reason why mice did not show panting preference.

## Methods

### Experiment 1

#### Mice

90 male C57/BL6j mice were used that were 8 weeks old when the experiment started. Food and water were freely available in the cages except for mice used for operant conditioning. Water was available for 3 hr after the behavioral experiment. Temperature was maintained at 24°C, and the light-dark cycle was reversed (12L:12D). All animals were treated in accordance with guideline of the committee of animal experiment in Keio. Permission number is 12079-(0).

#### Apparatus

A conventional conditioned place preference (CPP) apparatus for mice (ENV3015; MED) was used for the preference test and CPP experiment. The apparatus had three compartments: a side compartment with a grid floor and black walls (16 × 13 × 12 cm), another side compartment with a stainless steel mesh floor with white walls (16 × 13 × 12 cm), and a center compartment with a flat grey floor and grey walls (6 × 13 × 12 cm). The chamber was modified as follows. Walls and floor of the two side compartments were covered with the same grey acrylic plate so that the side compartments appeared similar. Each compartment contained a partition made of transparent acrylic that was placed 10 mm from the end wall. The center compartment was connected with the two side compartments by guillotine doors. Each compartment had a ceiling lamp. I used an iPod as the stimulus-presenting device and placed it at the end of each side compartment. The MED-SKED system controlled the experiment. White noise (75 dB) was broadcast throughout the experiment.

I used a touch screen operant chamber (Model 89541, Campden Instruments) for the operant conditioning experiment. The touch screen was located on the front panel (25 cm × 20 cm) and a liquid dispenser on the rear panel (5.5 cm × 20 cm). A panel with two rectangular windows (8 cm × 7 cm, separated from each other by 0.5 cm) was in front of the touch screen. The liquid dispenser provided 7 µl milk as a reward.

#### Stimuli

I took photographs of paintings by Renoir, Picasso, Kandinsky, and Mondrian (15 each) from art books and edited them for the slide show program in the iPod (see Table S1). Because the iPod screen is square, I trimmed some of the pictures to produce a square-shaped stimulus. The average luminance of the Renoir and Picasso paintings at the center of the chamber was 3.93 and 3.83 lux, respectively, a difference that was not statistically significant (*t*-test, *t*(15) = 0.23). The average luminance of the Kandinsky and Mondrian paintings was 5.59 and 6.04 lux, respectively, and this difference was also not statistically significant (*t*(15) = 0.78). The slide show program displayed 10 paintings in a random order every 10 sec.

#### Preference test

Thirty-two mice underwent six test sessions. Ten mice saw paintings by Mondrian in the left compartment and paintings by Kandinsky in the right compartment during the first three sessions; the sides were switched for the last three sessions. Ten additional mice underwent the test with the left-right order reversed. Another 6 mice saw paintings by Picasso in the left compartment and paintings by Renoir in the right compartment during the first three sessions; the sides were switched for the last three sessions. The remaining 6 mice underwent the test with left-right order reversed. The mice were placed in the center compartment, and the doors to the side compartments were opened 5 min later; the animal could then move around for 15 min. The floor and walls of each compartment were wiped with 70% ethanol after each test. The mean staying time in each compartment was recorded for analysis. Ten paintings of each artist were used for the preference test.

#### Conditioning

In the KM group (N = 20), morphine was injected in association with paintings by Kandinsky in 10 mice and injected in association with paintings by Mondrian in the other 10 mice. In the PR group (N = 12), morphine was injected in association with paintings by Picasso in 6 mice and injected in association with paintings by Renoir in 6 other mice. In the PR-G group (N = 14), morphine was injected in association with paintings by Picasso in 7 mice and injected in association with paintings by Renoir in 7 other mice. During the baseline, the mice were exposed to 10 Picasso and 10 Renoir, but each artist’s paintings were divided into 2 groups of 5. Five paintings of each artist were used for conditioning and remaining 5 painting were used in generalization test after the conditioning. This group received 4 days baseline training, 2 days for the paintings for the conditioning and 2 days for the paintings for generalization. The paintings for the generalization were never shown to the subjects during the conditioning. Mice in the mixed-PR group (N = 12) underwent CPP training with two sets of paintings. Both sets contained five paintings by Picasso and five by Renoir. Morphine was injected in association with one set for 10 mice and with another set for the remaining 10 mice. The conditioning procedure was identical in all groups except for the stimuli. Ten paintings of each artist were used for conditioning and another 5 paintings were used for generalization test in the KM and PR groups. Five of Picasso and Renoir were used for conditioning and another 5 were used for generalization test in PR-G group. These 10 paintings of each artist were shown in the baseline training. In mixed PR group, one set of stimuli consisted of one Picasso and one Renoir, and the other set consisted of another one Picasso and one Renoir.

Day 1 and Day 2: Each mouse was individually placed in the center compartment, and the doors to the other compartments were opened 5 min later; then, the animal was allowed to move around for 15 min. The staying time in each compartment on Day 2 was used as the baseline preference time. The floor and walls of each compartment were wiped with 70% ethanol after each test. The subjects in the PR-G group received more 2 days baseline training with paintings used for generalization test.

Day 3 to Day 8: Half the mice were given morphine injection on Days 3, 5, and 7 and saline injection on Days 4, 6, and 8. The compartment for drug administration was selected in an unbiased way. The other half of the mice received saline injection on Days 3,5, and 7 and morphine injection on Days 4,6, and 8. The mice were placed in the compartment immediately after the injection and kept there for 40 min.

Day 9: All mice underwent a post-training test that was exactly the same as the test used on Day 1 or Day 2. Their staying time in each compartment was measured for 15 min after restriction in the center compartment for 5 min.

Day 10: The mice underwent a similar test as on Day 9, but the stimuli were changed to new ones. Five paintings by Picasso and five paintings by Renoir were displayed to the RP group, and five paintings by Kandinsky and five paintings by Mondrian were displayed to the KM group. Five paintings of Picasso and Renoir never shown during the conditioning were displayed to the PR-G group. The mixed-RP groups did not undergo these tests.

#### Pharmacology

Morphine HCl (Dainippon Sumitomo Pharma, Osaka, Japan) was dissolved in physiological saline and administered i.p. The dose given was 3 mg/kg, and the volume administered was 10 ml/kg. Saline (10 ml/kg) was also injected i.p. After the injection, the animals were immediately placed into the CPP apparatus.

### Experiment 2

#### Operant discrimination

Four mice were used for the operant discrimination. After shaping the touch response to the screen, the mice were trained for pair-wise discrimination. A “free” reward (7 µl milk) was dropped onto the tray, and a nose poke to the tray started the first trial. Two images were presented on the screen. A touch on the correct image (Kandinsky) resulted in delivery of the reward, which was accompanied by a tone (3 kHz, 1000 msec). A nose poke into the tray turned off the tray light, and the intertribal interval (10 sec) started. If the mouse touched the incorrect image (Mondrian), no reward was given and the correction trial started. A correction trial consisted of re-presentation of the same set of stimuli. The correction trial was repeated until the mouse touched the correct image. The sides that were correct (S+) and incorrect (S−) were determined pseudorandomly. One session consisted of 20 trials or passage of 60 min. The criterion of discrimination was more than 85% correct choices during two successive sessions. When the mouse reached this criterion, discriminative training with a new set of stimuli began. This procedure was repeated four times with different pairs of stimuli. Finally, the mice received discriminative training with a pair of Picasso (S+) and Monet (S−) paintings

## Supporting Information

Table S1 **List of paintings.**
(XLS)
